# Supplementary material for: The methylglyoxal pathway is a sink for glutathione in Salmonella experiencing oxidative stress
Source: PLoS Pathog. 2023 Jun 2;19(6):e1011441. doi: 10.1371/journal.ppat.1011441 (PMC10266609; doi:10.1371/journal.ppat.1011441)
Supplement: S1 Text — Bacteria used in this study. Table B in S1 Text. Plasmids used in this study. Table C in S1 Text. Oligonucleotides used in this study. Fig A in S1 Text. Schematic representation and structures of metabolites of methylglyoxal pathway in Salmonella. Fig B in S1 Text. Transcription of methylglyoxal pathway genes. Fig C in S1 Text. Growth of ΔgloB Salmonella in glucose medium after complementation. Fig D in S1 Text. Effect of peroxide on Salmonella. Fig E in S1 Text. Glutathione buffering capacity in Salmonella lacking glyoxalase I. Fig F in S1 Text. Nicotinamide adenine dinucleotide content in ΔgloB Salmonella undergoing oxidative stress. (PDF) [file ppat.1011441.s001.pdf]

S1 Text

The methylglyoxal pathway is a sink for glutathione in *Salmonella* experiencing oxidative stress

Sashi Kant[1], Lin Liu[1,2], Andres Vazquez-Torres[1,2 *]

[1]University of Colorado School of Medicine, Department of Immunology and Microbiology, Aurora, Colorado-80045, USA; [2]Veterans Affairs, Eastern Colorado Health Care System, Denver, Colorado-80045, USA

*Andres Vazquez-Torres

Department of Immunology and Microbiology
University of Colorado School of Medicine
12800 East 19th Avenue MS 8333
RC1 North room P18-9131
Aurora, Colorado 80045, USA
Email: andres.vazquez-torres@cuanschutz.edu

Supplementary Materials and Methods.

H_2O_2 killing assays. *Salmonella* were grown overnight in LB broth or E salts minimal media [1.7 mM $MgSO_4$, 9.5 mM citric acid, 57.4 mM K_2HPO_4, 16.7 mM $(NH_4)NaHPO_4 \cdot 4H_2O$] supplemented with 0.4% D-glucose (EG) or 0.4% Casamino acids (ECA). Bacterial cells grown on LB broth were diluted to 5 x 10^5 CFU/ml in PBS. 200 μl of the bacterial cell suspension aliquoted per well of 96-well plates were treated with 400 μM H_2O_2 for 2 h. Cells grown in E salts minimal media were diluted in EG or ECA minimal media to 5 x 10^6 CFU/ml. Three milliliters of the bacterial cell suspensions were treated with 3 μl of a 250 mM H_2O_2 stock solution prepared in PBS. The specimens were incubated for 2 h at 37°C in a shaker incubator. Percent of *Salmonella* surviving the H_2O_2 treatment was calculated by dividing the CFU isolated 2 h after H_2O_2 treatment by the CFU recovered prior to the addition of peroxide.

Table A. Bacteria used in this study.

Strains	Relevant characteristics	Reference
Salmonella		
14028s	wild type of *S. enterica* serovar *Typhimurium*	ATCC
AV21184	Δ*mgsA*::Km [Δ*mgsA*]	This study
AV21186	Δ*gloA*::Km [Δ*gloA*]	This study
AV21031	Δ*gloB*::Km [Δ*gloB*]	This study
AV23044	Δ*gloB*::Km [pWSK29::*gloB*]	This study
E.coli		
DH5α	*supE*44 Δ*lacU*169 (ϕ80 *lacZ* ΔM15) *hsdR*17 *recA*1 *endA*1 *gyrA*96 *thi*-1 *relA*1	[1]
AV21196	WT-14028s [pfpv25::*roGFP2*]	This study
AV23043	DH5α (pWSK29::*gloB*)	This study

* Strains names briefly described in the text are indicated by square brackets ([ ]) next to their full names.

Table B. Plasmids used in this study.

Plasmid	Relevant characteristics	Source
pfpv25	*roGFP2*	[2]
pWSK29	low copy plasmid, *lacZ*α, Pn^r	[3]
pWSK29::*gloB*	pWSK29 + 756 bp *gloB* CDS with promoter, Pn^r	This study

Table C. Oligonucleotides used in this study.

Strains	Primer Sequence (5' → 3')
Δ*mgsA*::Km	**F:**ATGGAACTGACGACTCGCACCTTGCCGACGCGCAAACATACGGC AAACAAACCACCGCTG<br>**R:**TTATTTCAGGCGCTCGGCCAGATAACGCGCATAATCCGGATTAGA AAAACTCATCGAGCA
Δ*gloA*::Km	**F:**ATGCGTTTATTGCATACTATGCTGCGCGTCGGCGATTTGCCGGCA AACAAACCACCGCTG<br>**R:**TCAGTTACCCAGACCGCGGCCTGCGTCTTTAGCTTCAATCTTAGA AAAACTCATCGAGCA
Δ*gloB*::Km	**F:**ATGAATCTTAACAGTATTCCCGCGTTTCAGGACAATTACACGGCAA ACAAACCACCGCTG<br>**R:**TCAGAACGTGTCTTTCTTTGACCTTAACCATGCAAAACGCTTAGAA AAACTCATCGAGCA
**Plasmid**	
pWSK29::*gloB*	**F:** AAA *AAG CTT* TGA TTT CTG CGC TTA AAT TAC<br>**R:** AAA *GGA TCC* TCA GAA CGT GTC TTT CTT TGA
**qRT-PCR**	
mgsA	**F:** ACTGGGTGGAACGCCATCAG<br>**R:** AATGAGTGCGCCAACCTGCT
gloA	**F:** GGGGCGTTGAGAGCTACGAC<br>**R:** CTTTTACCGGCCCCGCTTCA
gloB	**F:** CGGCGGAAACGCAAGACAAG<br>**R:** AGCGTGTCGCCGCAGAATAA

* Restriction enzyme sites are italics.

REFERENCES

1. Hanahan, D. Studies on transformation of Escherichia coli with plasmids. *J Mol Biol* **166**, 557-580, doi:10.1016/s0022-2836(83)80284-8 (1983).
2. van der Heijden, J. et al. Exploring the redox balance inside gram-negative bacteria with redox-sensitive GFP. Free Radic Biol Med 91, 34-44, doi:10.1016/j.freeradbiomed.2015.11.029 (2016).
3. Wang, R. F. & Kushner, S. R. Construction of versatile low-copy-number vectors for cloning, sequencing and gene expression in Escherichia coli. Gene 100, 195-199 (1991).

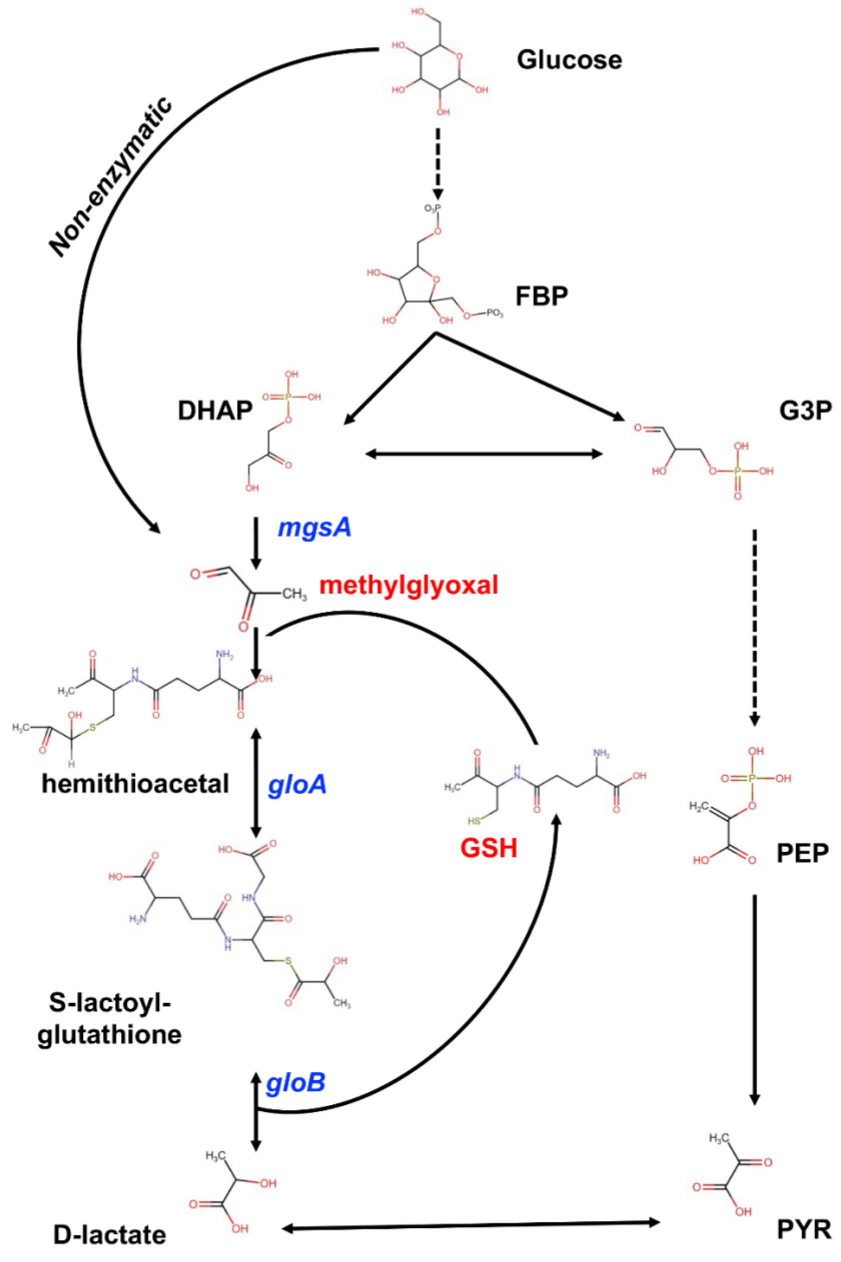

Fig. A. Schematic representation and structures of metabolites of methylglyoxal pathway in *Salmonella*. FBP, Fructose 1,6 bis phosphate; DHAP, Dihydroxyacetone phosphate; G-3P, Glyceraldehyde 3-phosphate; PEP, Phosphoenolpyruvate; PYR, Pyruvate; GSH, glutathione. Genes involved in the methylglyoxal pathway are shown in blue.

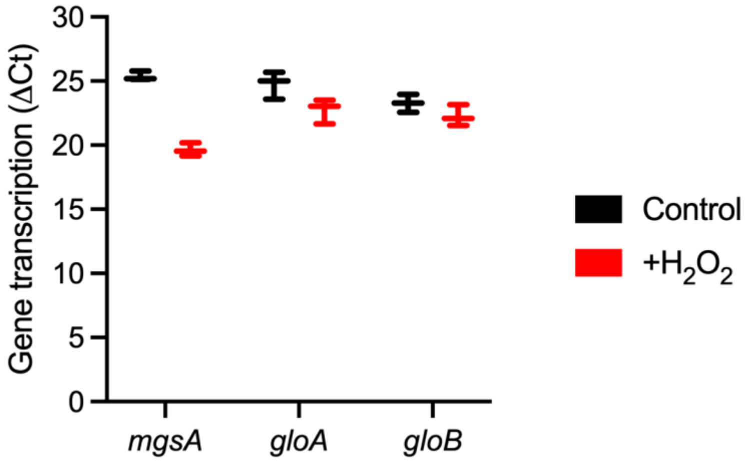

Fig. B. Transcription of methylglyoxal pathway genes. Gene expression analysis quantified by qRT-PCR of specimens isolated from *Salmonella* grown to an OD_{600} of 0.25 in MOPS-GLC minimal medium. Where indicated, bacterial cultures were treated with 400 μM H_2O_2 for 30 min before the RNA was isolated. The data, normalized to the *rpoD* housekeeping gene, are the Ct value ± S.D (N=3).

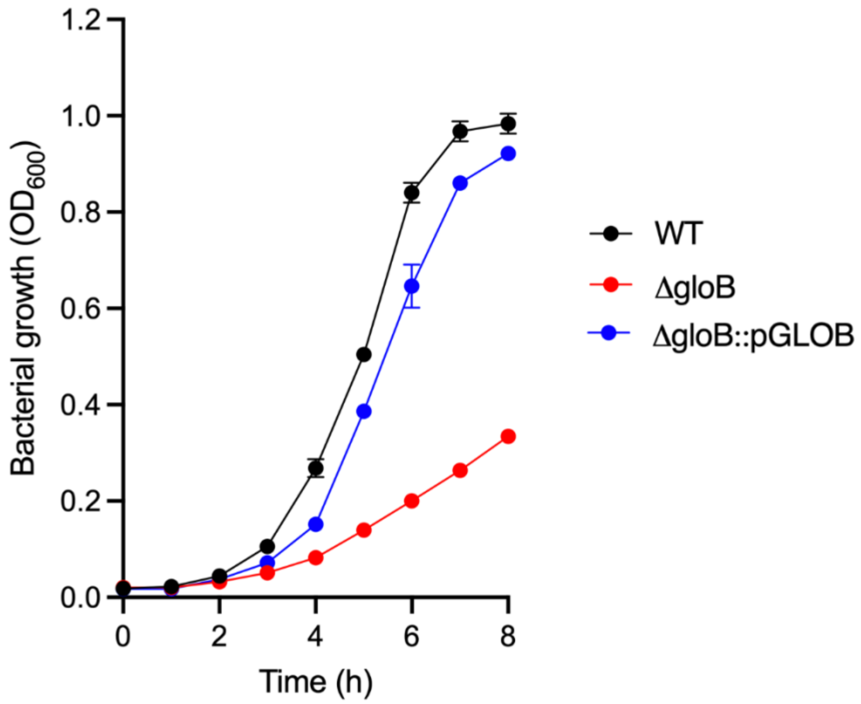

Fig C. Growth of Δ*gloB Salmonella* in glucose medium after complementation. Aerobic growth of *Salmonella* strains in MOPS-GLC minimum medium, pH 7.2, at 37ºC in a shaking incubator as assessed by OD_{600}. Δ*gloB* mutant was complemented with *gloB* gene expressed from the pWSK29 low copy plasmid. Data are the mean ± S.D (N=3).

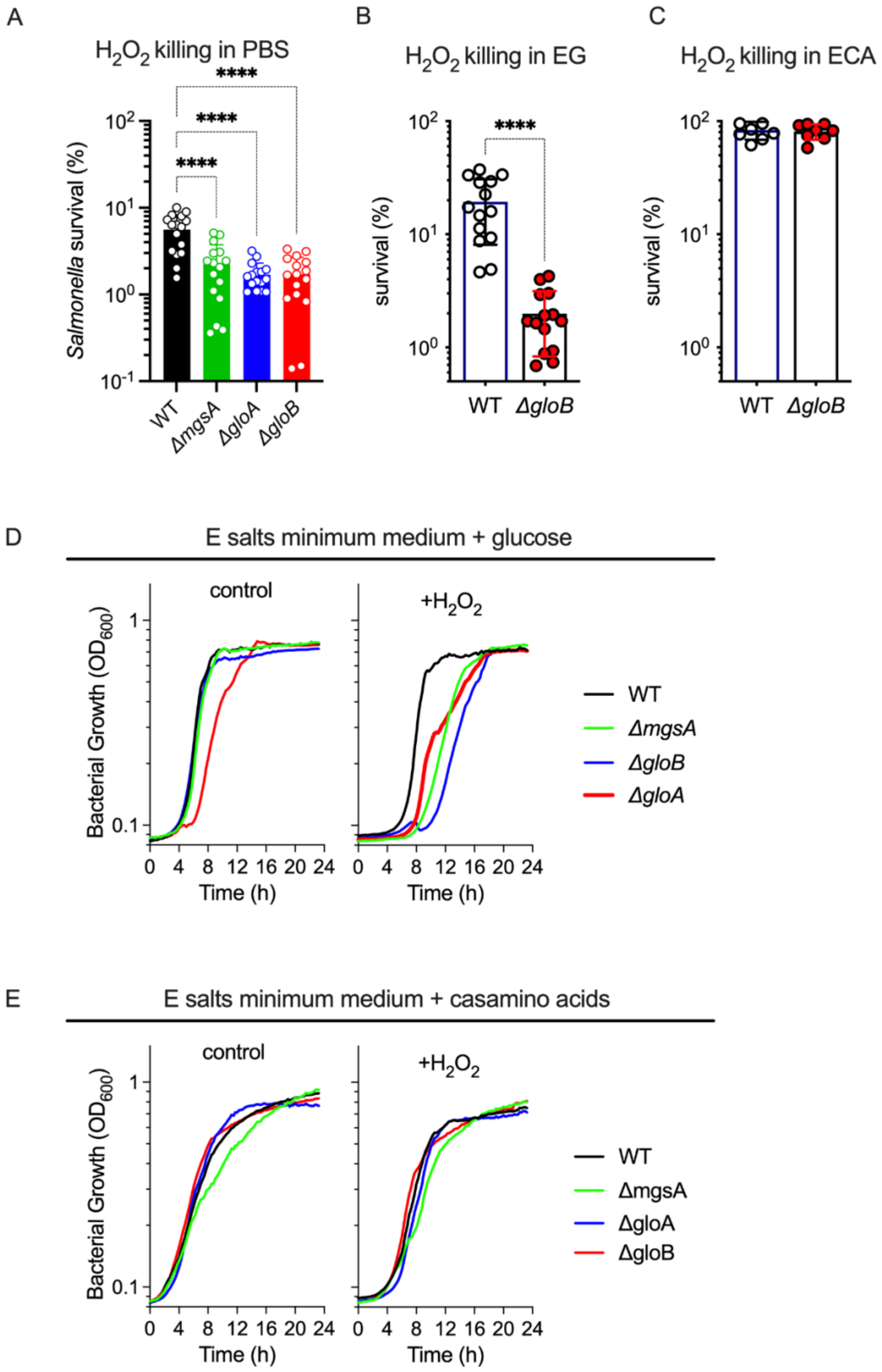
A
H_2O_2 killing in PBS
****
****
****
Salmonella survival (%)
10^2
10^1
10^0
10^{-1}
WT
ΔmgsA
ΔgloA
ΔgloB
B
H_2O_2 killing in EG
****
survival (%)
WT
ΔgloB
C
H_2O_2 killing in ECA
survival (%)
WT
ΔgloB
D
E salts minimum medium + glucose
control
$+H_2O_2$
Bacterial Growth (OD_{600})
1
0.1
0 4 8 12 16 20 24
Time (h)
WT
ΔmgsA
ΔgloB
ΔgloA
E
E salts minimum medium + casamino acids
control
$+H_2O_2$
Bacterial Growth (OD_{600})
Time (h)
WT
ΔmgsA
ΔgloA
ΔgloB

Fig D. Effect of peroxide on *Salmonella*. (A) Killing of *Salmonella* 2 h after treatment with 400 μM H_2O_2. Bacterial cultures grown overnight in LB broth were diluted in PBS to 10^5 CFU/100 μl/well/ 96-well plates. Some of the samples were spotted on LB plates to estimate bacterial input (T0). Where indicated, the samples were treated with 100 μl of PBS or 400 μM H_2O_2 prepared in PBS. After 2 h of treatment, the specimens were spotted onto LB agar. The percent of bacteria surviving H_2O_2 treatment was calculated in reference to bacterial numbers at time zero. Data are the mean ± S.D (N=16). ****, $p<0.0001$ as determined by one-way ANOVA. (B, C) Killing of *Salmonella* grown overnight and resuspended to 2.5 x 10^6 CFU/ml in E salts minimal media, pH 7.0, supplemented with 0.4% glucose (EG) or 0.4% Casamino acids (ECA). Where indicated, 3 ml cultures were treated with 250 μM H_2O_2 in 14 ml tubes at 37ºC in a shaker incubator. (D, E) The susceptibility of *Salmonella* to H_2O_2 was independently assessed by following bacterial kinetics of bacteria diluted from overnight cultures grown in EG or ECA minimal media and diluted in the same media to 5 x 10^5 CFU/well of 96 well plates. 100 μl volumes containing the bacterial suspensions were treated with or without 250 μM H_2O_2 prepared in minimal media. OD_{600} was followed in a Biotek Synergy H1 plate reader (Agilent, Santa Clara, CA) at 37ºC. Data are shown as the mean ± S.D (N=6-10).

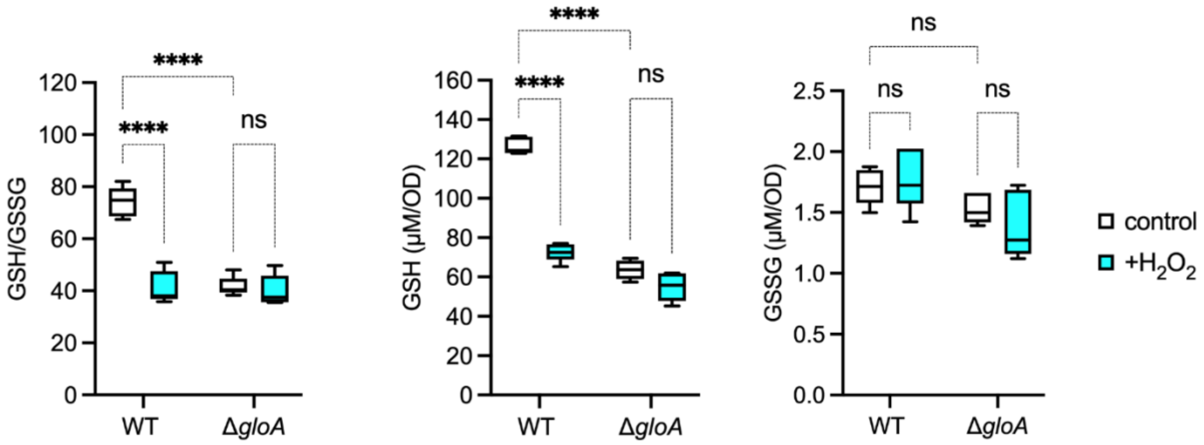

Fig. E. Glutathione buffering capacity in *Salmonella* lacking glyoxalase I. Estimations of reduced and oxidized glutathione (GSH and GSSG, respectively) in *Salmonella* strains grown to an OD_{600} of 0.25 in MOPS-GLC minimal medium, pH 7.2. Where indicated, bacterial cultures were treated with 400 μM H_2O_2 for 30 min prior to GSH measurements. Data are the mean ± S.D. (N=5) ****, $p < 0.0001$ as determined by two-way ANOVA.

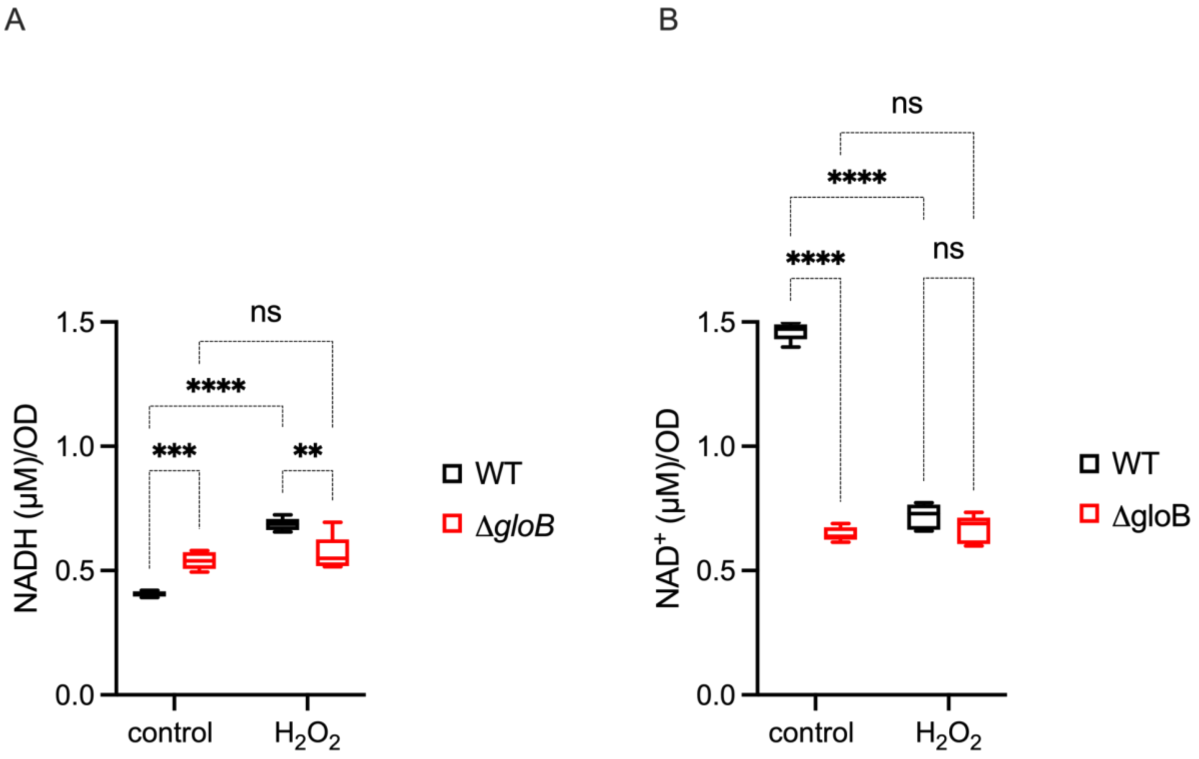

Fig F. Nicotinamide adenine dinucleotide content in Δ*gloB* *Salmonella* undergoing oxidative stress. A) NADH and (B) NAD^+ were measured in *Salmonella* strains grown in MOPS-GLC minimum medium, pH 7.2, at 37ºC to an OD_{600} of 0.25. Where indicated, bacterial cultures were treated with 400 µM H_2O_2 for 30 min. Data are the mean ± SD (N=5). **, ***, ****, $p< 0.01$, $p< 0.001$ and $p< 0.0001$, respectively, as determined by two-way ANOVA.
