# Supplementary material for: The methylglyoxal pathway is a sink for glutathione in Salmonella experiencing oxidative stress
Source: PLoS Pathog. 2023 Jun 2;19(6):e1011441. doi: 10.1371/journal.ppat.1011441 (PMC10266609; doi:10.1371/journal.ppat.1011441)
Supplement: Attachment — Submitted filename: Response.docx [file ppat.1011441.s002.docx]

**Responses to the Reviewer’s Comments.**

**Part II – Major Issues: Key Experiments Required for Acceptance**

Reviewer #1: N/A

Reviewer #2: 1. The depletion of GSH in the gloB mutant likely results in oxidation of proteins and enzymes in the ETC. This would explain the reduced oxygen consumption of the gloB mutant as well as the enhanced glucose uptake. This could be verified by showing increased lactate/acetate production of the mutant, which was shown to have a growth defect in glucose containing media.

Response: In response to these comments, we have measured L-lactate content in wildtype and Δ*gloB* *Salmonella*. As suspected by the reviewer, Δ*gloB* *Salmonella* makes higher amounts of L-lactate than wildtype controls. Furthermore, L-lactate production is increased in *Salmonella* following H_2_O_2_ stress, consistent with the idea that oxidative stress favor overflow metabolism. These data are shown in revised **Fig. 6** panel D.

2. Figures 1, 4 and 5B: The scale for the GSSG levels should be changed so we can appreciate the levels in the different conditions.

Response: As recommended, the scale for the GSSG measurements has been changed for easier appreciation of the levels of oxidized glutathione in the samples.

3. Chemical structures in Figure 2 would be ideal.

Response: The chemical structures are shown in revised **Fig. S1**.

4. Figure 3C should reflect the level of transcript not just fold induction. Do gloA and gloB show low induction because they are already abundantly expressed?

Response: Representation of transcription levels for each of the conditions is shown in **Fig. S2** supplementary files. These data show that H_2_O_2_ induces higher levels of *mgsA* gene transcription compared to *gloA* and *gloB* genes.

5. Lines 131, 151 and throughout. The use of “methylglyoxal mutants” is improper. Perhaps “mutants in genes encoding enzymes of the methylglyoxal pathway” or “methylglyoxal pathway mutants”.

Response: As suggested, we now indicate methylglyoxal pathway mutants or mutants in genes encoding enzymes of the methylglyoxal pathway.

6. Lines 77 and 236. Another word other than “cranked up” should be used for increased flux.

Response: As indicated, the verb has been changed.

7. Line 238-39. Reference 28 is not appropriate to support this statement. There are several reviews on phosphosugar detoxification in bacteria.

Response: As suggested, a newer review on phosphosugar detoxification in bacteria has been included. We have also clarified that methylglyoxal has been associated with the formation of advanced glycation end-products in humans.

Reviewer #3: (No Response)

**Part III – Minor Issues: Editorial and Data Presentation Modifications**
Please use this section for editorial suggestions as well as relatively minor modifications of existing data that would enhance clarity.

Reviewer #1: - Did the authors evaluate the concentration of GSH in a gloA mutant in the presence or in the absence of H202 stress ? Because a gloA mutant should be as defective as a gloB mutant for the GSH recycling as the GSH is linked in the hemithioacetal molecule after the spontaneous reaction between GSH and methylglyoxal, before the intervention of gloA and gloB enzymes. That would not change the conclusion about the fact that the gloA mutant is able to grow on glucose because of other possible redundant proteins, but that would confirm that gloA is as important as gloB in this new H202 resistance mechanism. Depending on the results, the sentence lines 137/138 could be nuanced. This experiment could be interesting especially because there is a different phenotype in WT BMDMs (fig 7 A) between the gloA / mgs mutants and the gloB mutant, phenotype which is restored at the same level in Cybb-deficient BMDMs. This difference could be discussed as it is not so obvious why gloA and gloB have a different phenotype in WT BMDM.

Response: As suggested, we have now measured GSH/GSSG in Δ*gloA* mutant in presence or absence of H_2_O_2_. The results are shown in **Fig. S5**. The GSH and GSSG content is very similar between Δ*gloA* and Δ*gloB Salmonella* in the presence or absence of peroxide stress, consistent with the idea that both glyoxalase I and glyoxalase II are involved in the adaptation of *Salmonella* to oxidative stress.

- Did the authors try to measure the concentration of the methylglyoxal molecule itself in Salmonella in the presence or in the absence of H2O2 using a kit for example?

Response: As suggested by the reviewer, we have now measured methylglyoxal production in *Salmonella* following oxidative stress. The results, which are consistent with the aldehyde estimations, are shown in **Fig. 3B**.

- Fig 7A: The method used to evaluate the bacterial burden in BMDMs after infection seems different between the time 0 and times 2 and 18 hours. Indeed, gentamycin treatment has been used only for T2 and T18, but not for T0. How to be sure that the bacterial burden at T0 correspond to the intracellular bacteria only? Did the authors washed several times the wells with PBS? Even if the results are expressed in percentage, using the same method for all timings will maybe avoid this high difference of bacterial burden between T0 and T2h. It could also be nice to show the results as “CFU/mL” with the absolute values for each timing (T0, T2 and T18), and not using a percentage.

Response: The reviewer is correct: the specimens collected at time zero had not been treated with gentamicin. As suspected by the reviewer (and to eliminate a significant proportion of extracellular *Salmonella*), the specimens collected at time zero were washed with prewarmed PBS prior to the lysis step. We followed a published protocol (PMID: 18433645). This information has been presented more clearly in the Materials and Methods section. As requested, we have shown the data as CFU/ml.

- Fig 7B and C: Did the authors weight the organs before plating? That would allow to express the CFU per gram of organ, normalizing then all the data. If not, could the authors at least mention that they did (or not) observed any splenomegaly or hepatomegaly?

Response: The organs were not weighted before plating. No splenomegaly or hepatomegaly were observed at the time of euthanasia. This information has been added to the revised document.

- The GSH and its canonical mode of action to scavenger H202 should be briefly introduced and described in the introduction as the experiments looked at the concentrations and ratio of GSH/GSSG in order to highlight another, new mode of action of GSH in the peroxide resistance.

Response: As recommended, a brief introduction on the canonical role of GSH as a scavenger has been introduced in the results section presenting data of **Fig. 1**.

- The protocol for the qRT-PCR done for the Fig 3C is missing in the “Materials and Methods” section. Please add the protocol in this section and explain the analysis method used.

Response: Sorry for the omission. This information has been added to the Materials and Methods section.

- Please, go through the manuscript to check that all the abbreviations are defined the first time they are used. For examples: “MOPS” (line 95), “Reactive oxygen species (ROS)” (line 62), glutathione (GSH) (line 84), “MOPS minimum medium containing glucose as sole carbon source (MOPS-GLC) (line 96), “DTT” (line 96), “MTT” (line 355), …

Response: As suggested, we have defined the abbreviations the first of time of usage.

- Instead of saying “loss of GSH” (line 112), the authors should mention the “decrease of GSH concentration” as GSH is still present in the condition where Salmonella is treated with H202 (Fig 1B).

Response: As indicated, the change has been made.

- The authors should modify the following sentence “Reaction of methylglyoxal with GSH forms the S-lactoylglutathione conjugate” (line 116) as the first product formed after the reaction between methylglyoxal and GSH is the hemithioacetal, as showed in Fig 2.

Response: As indicated, the language has been revised.

- The “in PBS” (line 148) from “Salmonella grown to stationary phase in LB broth when exposed to H2O2 in PBS“ is a bit confusing. It should be either removed as it is never used somewhere else or clarified if the authors want to say that H2O2 is diluted in PBS. Also, if it is the case, it should be mentioned in the “Materials and Methods” section.

Response: The methodology for testing killing of *Salmonella* by H_2_O_2_ has been presented more clearly in Results and Materials and Methods in main text and Supplementary Information.

- Fig 1B, 5B and 5C: the scale of the Y axis should be modified in order to visualize that there is indeed no significant difference (Fig 1B and Fig 5C) or a significant difference (Fig 5B) in the GSSG concentration.

Response: As requested, the scale for **Figs 1B**, **5B** and **5D** has been changed for easier visualization.

- Except if it is a double direction reaction, which does not appear on the Fig 2, the methylglyoxal cannot be detoxified by the methylglyoxal synthase (lines 240-241), as this enzyme is the one forming the methylglyoxal from DHAP. Please correct/precise.

Response: As requested, we have clarified that methylglyoxal is detoxified by its reaction with glutathione, not by methylglyoxal synthase.

- The use of the “greAB” mutant of Salmonella is described in the “Materials and Methods” section (line 318), but then never mentioned in the manuscript. Please, either modify the “Materials and Methods” or clarify the use of this mutant in the “Results” section.

Response: Sorry for the error. Mention to the *greAB* mutant has been erased from the revised manuscript.

- “Salmonella” should be in italic (line 598).

Response: As suggested, *Salmonella* is shown in italics.

Reviewer #2: See above

Reviewer #3: Line 49: …“that is activated as overflow metabolism is favored during periods of oxidative stress” Run-on sentence? This is difficult to understand.

Response: As suggested, the sentence has been revised.

Reference 23: A more up-to-date review could be cited here, PMID: 34585982
Response: As indicated, the reference has been added to the revised manuscript.

Line 143: If I understand this correctly, the idea for explaining the gloB mutant phenotype is: Methylglyoxal, produced in large quantities from glucose degradation, reacts and thus depletes GSH. In the gloB mutant, S-lactoylglutathione accumulates, and S-lactoylglutathione acts as a GSH sink. It might be helpful for the reader to point that out as a main conclusion at the end of the paragraph.

Response: As suggested, this information has been added at the end of the paragraph.
